# Visual search patterns for multilingual word search puzzles, a pilot study

**DOI:** 10.16910/jemr.16.1.6

**Published:** 2023-03-31

**Authors:** Tanya Beelders

**Affiliations:** University of the Free State, Bloemfontein, South Africa

**Keywords:** Eye movement, eye tracking, word search puzzle, search strategy, gaze

## Abstract

Word search puzzles are recognized as a valid word recognition task. Eye gaze patterns have been investigated during visual search and reading, but the word search puzzle requires both searching and word recognition. This paper will discuss findings from an eye-tracking study of word search puzzles in three languages, of varying fluency for the participants. Results indicated that participants employ a search strategy that is somewhat dependent on language fluency and varies from a rigid, structured search pattern to randomly searching for a target word. The majority of gaze measurements are not significantly influenced by either word length or fluency of presented language, although mean fixation durations are longer for shorter words.

## Introduction

This study is interested in visual search for words through the medium of a word search puzzle. A word puzzle has target or goal words that must be found in a grid that is filled with letters. The puzzle will therefore not have any features that are more salient than surrounding features as it consists of ordered rows and columns of letters, some of which form words. In order to find a target word, a person must perform a visual search of the puzzle, but with no salient features. Since it contains letters it requires reading or word recognition to occur. The word puzzle will be presented in different languages in order to investigate language fluency on the visual search pattern.

Visual search and reading are often the foundation or variable under investigation in eye movement research. Therefore, there is a wealth of information regarding eye movements during reading, such as the types of eye movements that are exhibited, typical duration of fixations during reading and how to determine, using gaze measures, when a reader is experiencing difficulty with the material (14) as well as differences in gaze measures for bilingual readers (see Abdel Latif, 1, for a detailed summary), including in a South African context (4). Similarly, tracking gaze during various visual search tasks has been under investigation for many years. In his seminal work, Yarbus (19) demonstrated that the task of the viewer heavily influences the gaze patterns that are used. In particular, the search pattern can be executed in either a top-down or bottom-up procedure, where top-down is governed by voluntary selection of features and bottom-up search is governed by automatic viewing of salient features in the image (5, 6; 17). Since this study aims to investigate gaze patterns on word search puzzles, it is relatively unknown what gaze patterns will emerge, since the underlying task is a visual search in the absence of salient features that requires some competence in reading.

## Background

Many languages have spaces between words while some do not use spacing between words. Either way, when reading, the reader must be able to identify words in the text. This process is called word segmentation – the identification of the start and end of individual words (3; 16). During reading, the eye moves from word to word, and Rayner (14) has shown that the preferred initial fixation when reading English is the center or slightly to the left of center for each individual word. Hence, when moving the eye using a saccade, the goal of the reader is to end the saccade to the left of the middle of the word to be read.

When reading a language such as English that has spacing, there are in fact two spacing characteristics that influence eye movements, namely intraword (spacing between letters within words) and interword (spacing between words) spacing. Interword spacing can have a large effect on saccade target selection and removing the spaces completely between words can slow reading down by 35% as it disrupts the word segmentation process (8; 14). More and longer fixations are evident when both intraword and interword spacing are decreased (10) but merely adjusting the intraword spacing does not affect reading speed or comprehension (11). Fixation durations are shorter when intraword spacing is decreased and interword spacing is increased and this type of spacing causes delays in word recognition when reading for comprehension (16). However, when only intraword spacing is decreased, fixation durations are longer for all English children readers while dyslexic children have shorter fixations when intraword spacing is increased (11) and saccade targeting is negatively affected as well (2). On the other side, in adults the number of fixations increases and fixation durations are decreased when extra spacing between letters is introduced (13). In written languages that do not have spaces, introducing spacing between words increases word identification (15). Saccade targeting places the gaze roughly in the middle of the word when spacing is introduced (9). Our study introduces increased spacing between letters, but also, since this is not a reading task, there is no delineation between words and words are surrounded by random letters and not by other meaningful words.

Similar studies have been conducted on word recognition and word search. For example, eye movement analysis during visual search of word lists indicates that fewer fixations and smaller saccades are required for a vertical list but that fixations are longer (12).

A word search puzzle is a proven method to study word recognition in bilingual individuals (18). When using this method, it was found that L1 (in this case Dutch) words were recognized more frequently than L2 (English) words but that the proficiency in L2 did not influence recognition (18).

The underlying supposition of this study is that viewers may undertake a visual search of a puzzle in one of two ways, namely either an almost random search of the puzzle looking for a letter contained in the word being searched for or even the word itself that could be seen at a single glance. The second strategy might be to employ a more structured search for the word by looking, for example, letter-by-letter from the top left corner to the bottom right corner. This supposition was previously confirmed by (7) who tested 13 participants completing simple word search puzzles. Some participants used a rigid search pattern while others completed the puzzle more haphazardly. Furthermore, they found that those using a non-rigid search pattern completed the puzzle faster than those using a rigid pattern. This study will seek to determine whether these search patterns are indeed used to find a word in a word puzzle. Furthermore, by presenting puzzles in different languages, including a language that is not familiar to the participants, the study will investigate whether the search pattern employed in the first language (L1) puzzle is replicated in an unknown or second language (L2) puzzle or if it is abandoned for a different strategy.

A second point of interest is to determine whether it is easier to find a word in a known language than a language that a participant is not fluent in. It is suspected that finding a word in an unknown language would take longer as the word will not be recognizable but will have to be verified letter-by-letter. In a known language the word should be recognizable and require less letter-by-letter inspection.

## Methods

### Participants

The gaze movements of thirteen participants (9 males and 4 females) were captured during testing. All participants were staff members of the university where the study was conducted and were personally approached to participate in the study. All had normal or corrected-to-normal vision. The average age of the participants was 37 years of age. All participants were fluent in both English and Afrikaans, being able to fluently read, write and speak both languages, while a single participant was also fluent in Sesotho. In this instance, Sesotho was the second language of that particular participant, while only one other participant was a first language English speaker. Sesotho, or Southern Sotho, is an African language, in particular one of the 11 official languages of South African, and is spoken by many Africans living in the Free State province of South Africa.

It is acknowledged that the small sample size is a limitation of the study, however, the intention is to perform a repeated measures ANOVA as participants all conducted a search on 6 puzzles.

### Procedure

The stimuli used were word search puzzles. Two puzzles in each of the testing languages, namely English, Afrikaans and Sesotho were presented to the participants. For each language, there was a short word that had to be found and the second puzzle was a longer word. Therefore, participants searched for a long and a short word in their first language (L1), second language (L2) – both of which they were fluent in – and third language (L3), in this case a language they were not fluent in. The puzzle contained no other words in the presented language – hence no other distractors were presented as hidden words. Each puzzle had the same font size and spacing between letters. Spacing was increased between letters to ensure more accurate eye tracking. Participants viewed the puzzle until they found the word they were searching for, at which time they could click on the start and end letter of the word in order to identify that the correct word was found. The order the puzzles were presented in was counterbalanced. The orientation (vertical or horizontal) and position of the target word was randomly selected when the puzzle was generated but every participant received the puzzles with the same orientation and position. Target words were only top-to-bottom or left-to-right, hence no target words were presented in reverse order.

### Hardware

Gaze data was captured using a Tobii T120 eye-tracker. The stimuli was presented on the screen with a resolution of 1920x1080 and participants were seated approximately 60cm from the screen. The data capture rate of the T120 is 120Hz and the velocity-based Tobii IV-T algorithm was used to identify fixations.

### Measures

A number of standard eye-tracking measures were analyzed in order to determine if there was a difference in behavior between puzzles presented in different languages and with varying target word lengths.

Time to first fixation is measured in terms of how long before the participant first fixated on the target word. This measure will give an indication of the amount of time taken to locate the target word. This metric is compared to the time it then took the participant to click on the target word as a means to identify and indicate that they had found the correct word. These measures together will clarify how long before the word is found and how identified as the correct target word.

The duration of the first fixation on the target word was also analyzed under the assumption that it might differ between languages and target word lengths. The measure will give an indication of the duration of the fixations required in order to verify that the target word has been found.

Mean fixation duration is a standard measure of gaze analysis, allowing researchers to determine whether there is increased cognitive load or difficulty being experienced during, for instance, a reading task. Since the word search is a visual search of text, this measure could shed light on the cognitive load required to locate a word in various languages and of various lengths.

The final fixation measure analyzed was the number of fixations made during the search process. This will show whether there were many or few fixations required to find the target word. A relationship could exist between the number of fixations and the search strategy employed as well as the language, since an unknown language might require more fixations to find the target word.

Saccades are another measure used to distinguish between levels of cognitive load as well as whether a top-down or bottom-up search strategy is being used. Since this is a visual search in the absence of salient features, the saccade amplitudes will shortly be discussed as it is surmised that the search strategy heavily influences saccades.

### Analysis

Since all participants completed all 6 puzzles, two in each language, a repeated measures ANOVA was used to analyze the gaze measures. Owing to the small sample size, a power analysis is also reported to ensure any conclusions drawn are done so with the power of the analysis taken into cognizance. In terms of identifying the gaze patterns, this was done manually.

Viewers of word puzzles use either a random search pattern or a more rigid search pattern, moving from letter to letter in a structured way until the desired letter is found and inspected to determine whether the target word has been found. The employed search strategy was determined through manual visual inspection of each gaze plot for the duration of the search. Each puzzle, per participant, was then designated as being solved using either a random, structured or semi-structured search pattern using the following criteria:

- Structured search patterns are one where a very distinctive pattern is seen whereby the participants move from letter to letter either horizontally, row-by-row or vertically, column-by-column. This type of pattern is similar to what one would see for a typical reading task.
- A random search is one where the participant can clearly be seen to be “jumping” to random positions in the puzzle and doing a letter-by-letter search. This could be similar to visual search of a scene in free viewing and in the absence of obvious salient features.
- The semi-structured is then a combination of the two afore-mentioned search patterns. With this strategy there will be clear snippets where a structured letter-by-letter search is used interspersed with random jumps to various places on the search grid.

## Results

### Search pattern

**Figure 1:**
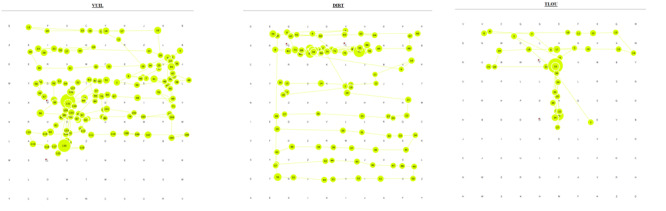
Gaze plots of a single participant who employed a structured search pattern for all three languages (showing only short words)

**Figure 2:**
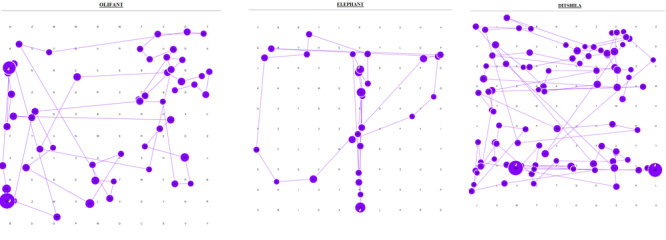
Gaze plots of a single participant who employed a random search pattern for all three languages (showing only long words)

The gaze plots in Figure 1 show a single participant using a structured search pattern and Figure 2 a single participant using a random search pattern.

**Figure 3:**
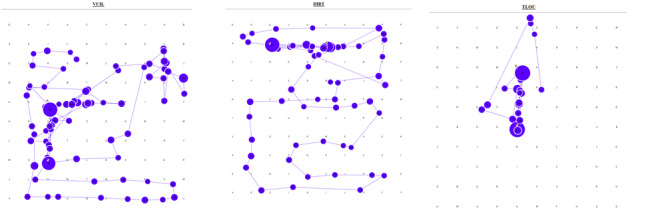
Gaze plots of a single participant who employed a semi-structured search pattern for all three languages (showing only short words)

Furthermore, in this study, it was also seen that some participants alternated between the two search strategies (Figure 3) in a single puzzle, using what will be called a semi-structured search strategy.

**Figure 4:**
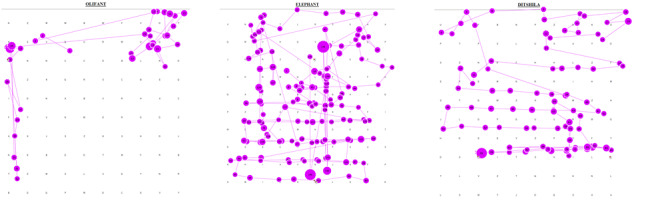
Gaze plots of a single participant who moved from random to structured search as the proficiency in the search language decreased (showing only long words)

Since word searches were presented in multiple languages, it was also anticipated that participants might change their search strategy based on their knowledge of the language. This was seen in isolated cases where participants would search randomly in some languages and rigidly in others (Figure 4).

**Figure 5:**
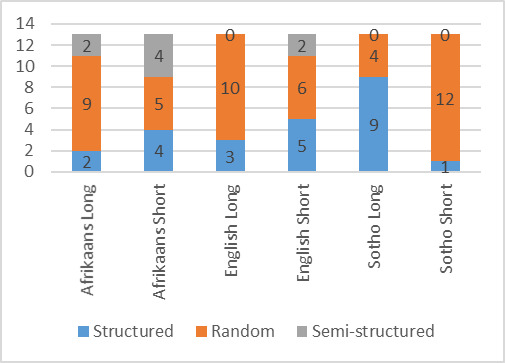
Number of participants using each of the search strategies

The graph in Figure 5 shows the number of participants who employed respectively structured, random and semi-structured search strategies for each word puzzle. As can be seen, the numbers varied as participants adapted their search strategy to the current puzzle. Overall, the majority of participants preferred a random search for the target word. However, as the fluency in the language decreased, where English was the majority L2 and SeSotho the majority L3, the number of participants employing a structured search pattern increased.

### Power analysis

Power analysis was conducted to determine the statistical power of the results. Assuming RMSSE to be 0.3, the power of the statistic is calculated as 0.7, below the desired level of 0.8. Hence, statistical results will be reported with the view that the study is underpowered.

### Time to find target word

**Figure 6:**
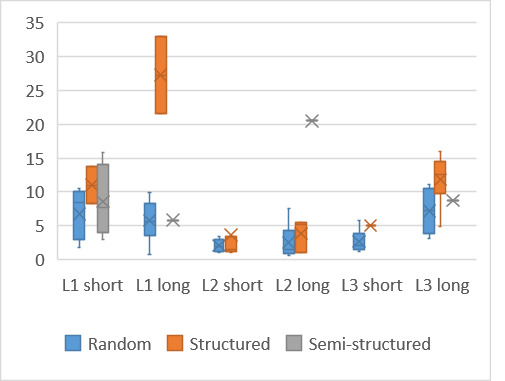
Time to first fixation on target word

The time to the first fixation on the target word was in general shorter when participants used a random search strategy (Figure 6). This was seen for all three languages L1, L2 and L3 and confirms prior findings (7) that a random search yields results faster. Interestingly using a structured search pattern for a long word in L1 resulted in a long time to first fixation, much longer than the other search patterns.

The majority of participants had an L1 of Afrikaans and the long word was placed high in the puzzle, similar to the English puzzle, hence it should not have taken markedly longer to locate the target word. Inspection of the gaze plots shows that two participants clearly did not see the word on their first pass, somehow skipping past and then finding the word on a subsequent pass. This could be due to the fact that the word was in the first column – the participants either did not see the starting letter or negated to search the first column, concentrating instead on the center and right of the puzzle first.

Since all participants viewed all word puzzles, a repeated measures analysis was conducted to determine whether there was a difference in times for the participants as their fluency decreased for the presented puzzle. In this case, there was a significant difference between the time taken to fixate on the target word (F(5, 50) = 3.7, P < 0.05). On average and regardless of search strategy, participants took the longest to fixate on the target word in their L3 language (long), followed by L1 (long) and L1 (short).

After participants had located the word, they were asked to click on the start and end letter as a means of identifying the word and ending the puzzle. The time to first fixation and the time to correctly identify the word by clicking on it should differ. Hence the time to identify the word by clicking on it was also analyzed.

**Figure 7:**
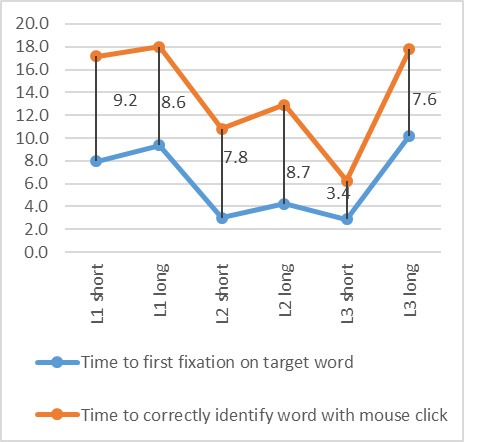
Difference in times between first fixation on target word and correctly identifying it (seconds)

A repeated measures analysis showed a significant difference in the time to correctly identify the target word, F (5, 60) = 2.7, p < 0.05. On average, participants took longer to identify the correct word in their L1 and the long word in their L3 (Figure 7). The difference in time between first fixating on the word and then correctly clicking on it is fairly steady for all puzzles, apart from the short word in L3 which has a much faster response time to click on the target word.

Using the mouse click as the time it takes to verify that the target word has been found, it can be deduced that it took between 6.2 and 18 seconds for participants to correctly identify the words.

### First fixation duration on target word

**Figure 8:**
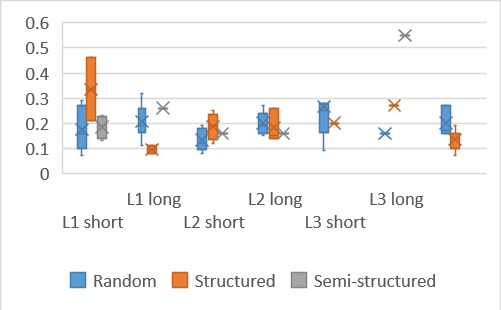
First fixation duration (seconds) on target word

The duration of the first fixation on the target word was similar for all word puzzles (Figure 8).

The repeated measures also showed that there is no significant difference between the first fixation duration for the various word puzzles (F(5, 50) = 1.4, p > 0.05).

### Mean fixation duration

**Figure 9:**
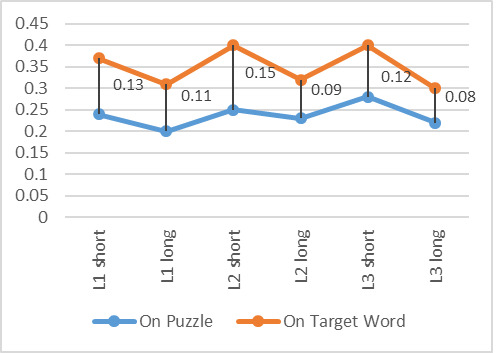
Mean fixation duration on target word and for duration of search on whole puzzle

Mean fixation durations on the target word were similar across the various puzzles, both in terms of language and target word length. Similarly, there were minor fluctuations in the mean fixation duration between the puzzles (Figure 9) during the search to locate the target word.

There was no significant difference between the mean fixation duration on the target words between the various puzzles (F(5,50) = 1.9, p > 0.05). However, a significant difference was found between mean fixation durations on the whole puzzle, F(5, 60) = 5.8, p < 0.05, indicating that participants were affected by the puzzle. Interestingly, mean fixation durations were longer when searching for the shorter words. Understandably, the durations also increased as fluency decreased. The increased duration for shorter words indicates more difficulty when searching, or perhaps participants attempted to look for the whole word with a single glance, thus increasing fixation durations.

### Number of fixations

**Figure 10:**
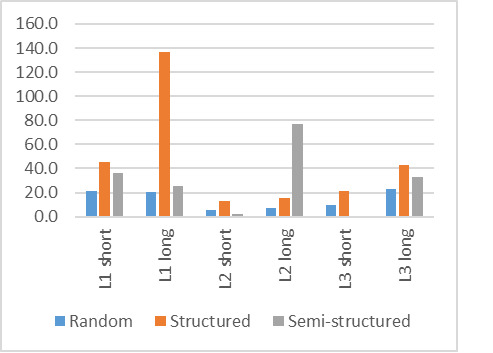
Number of fixations before target word was fixated on

The number of fixations before the target word was fixated on (Figure 10) were similar for all words and all search strategies, apart from L1 long (structured) and L2 long (semi-structured), which had a large number of fixations before.

A repeated measures ANOVA showed that the number of fixations was significantly different (F(5,50) = 3.1, p < 0.05).

**Figure 11:**
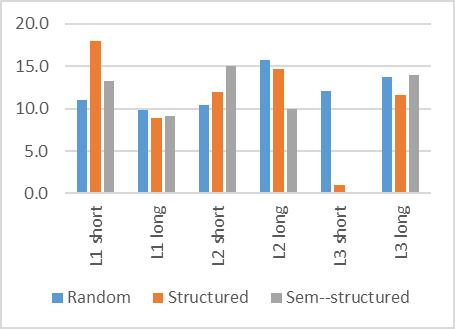
Number of fixations on target word

The number of fixations on the target word (Figure 11) was similar for all instances. In the case of this metric, the amount of time required to verify the target word had been found will influence the number of fixations. It could be expected therefore, that the longer words would have more fixations but inspection of the graph does not indicate a large disparity between long and short words.

The number of fixations on the target word was not significantly different for participants (F(5,50) = 1.4, p > 0.05).

### Number of visits to target word

**Figure 12:**
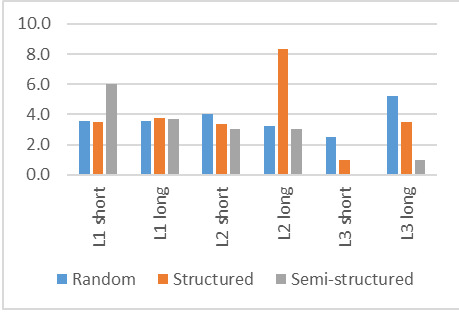
Number of visits to target word

The number of times a participant returned to the target word (visits) is shown in Figure 12. Only L2 long had a large number of visits to the target word. It could be expected that the need to confirm that the word had been found will influence this metric and that it would thus be higher for a language of lesser fluency but this does not appear to be the case.

### Saccade amplitude

Saccade amplitude is most likely dependent on the search strategy, with random search strategies eliciting the longest amplitudes and structured the shortest. This is indeed the case, with random searches having amplitudes larger than 4, and semi-structured and structured mainly having amplitudes less than 3.6.

If the search strategy is disregarded, then the search for the short word in L1 elicited the shortest saccades (3.5), while the remainder of the searchers fluctuated in the range between 4 and 4.4.

## Discussion

Three different search strategies were identified, namely structured, semi-structured and random. The majority of the participants chose to use a random search pattern in order to identify the target word. This confirms prior findings where participants were seen to use either a haphazard searching method or a more rigid, structured method (7). However, in the current study some participants alternated between search patterns, with more participants opting for a structured search pattern as their fluency in the presented language decreased. Therefore, as the participant became less comfortable in the language they adopted a more formal search process. This was evidenced by the number of participants who were evaluated to be used a structured search pattern increasing in L2 and further increasing in L3. Word recognition is dependent on the fluency of the language and the change in search pattern leads one to believe that the participant makes a conscious decision to change searching behavior as they realize that the word they are searching for is unfamiliar. However, as in previous studies (18), the proficiency of the language is not an inhibiting factor in identifying a target word in a word puzzle.

The search pattern could heavily influence the time to identify the target word. For instance, if the target word were near the bottom of the puzzle, a structured search, starting at the top of the puzzle, could very well significantly increase the time to find the correct word. Even in this case, where the puzzles were not large and the positioning of the target word was similar, a random search was more efficient than a structured search. This confirms the findings of Harrel et al., (7), who also found a random, or haphazard search to be more efficient. The interplay between the search pattern and language fluency is therefore concluded to have an impact on efficiency as the search pattern changes and word recognition will be slower in a less fluent language.

Finding and recognizing the word was the first part of successfully completing the puzzle. The participant had to then click on the word to verify that they had found it. Using the mouse click as the time it takes to confirm that the target word has been found, it can be deduced that it took between 6.2 and 18 seconds for participants to correctly identify the words. This is similar to the seek time found by Haskell et al. (2017) who found a mean seek time of 16.7 seconds when distractors were present but markedly faster than the seek time of 30.5 seconds when no distractor words were present. The shorter seek time in the absence of distractors, however, makes more sense since there is only a single word to recognize and other groupings of letters can easily and quickly be discarded as nonsensical. Therefore, the participant does not “waste time” as it were reading and recognizing other words which are not the target word.

In terms of gaze metrics, most were not significantly different between the participants, either in terms of language or word length, showing that even though fluency in language decreases it is still possible to maintain an efficient search. First fixation durations are by and large shorter than typical reading fixations of 225-250ms as found by Rayner (14). This corresponds to previous studies that found fixations to be shorter when intraword spacing is increased (10; 13). Spacing of rows and columns could thus play a vital part in word search puzzle completion times. Since there was no delineation of words in the current study but also there were no surrounding words and the study does not require reading, the number of fixations and effect of interword spacing on word recognition cannot be extrapolated in this case.

However, it can be concluded that mean fixation durations are slightly shorter than those typically found during reading English text (14). The conclusion here would be that the participant does not have to read and assimilate a whole word but rather perform word recognition based on a minimum of two letters. The mean fixation duration on the target word itself is, on average, longer than on the rest of the puzzle, as this could be a process of prolonged word recognition and verification that the correct word has been found. Mean fixation durations were longer for shorter words indicating that participants possibly tried to identify the shorter words using a different strategy than the longer words, namely less but longer fixations in order to assimilate or view all the letters of the word in a single glance.

The number of fixations before the target word was located were similar for all puzzles. The position of the word could have a strong influence on this metric under normal circumstances. However, the puzzles used were small and word placements were roughly in the same position in terms of how far “down” in the puzzle they were placed hence word placement should not be overly influential in this study. The number of fixations in this instance is also not comparable to those when reading a passage or text.

## Conclusion

The present study is considered to be a pilot study with a small number of participants. This makes it difficult to generalize to the wider population. Although the number of participants is the same as in similar studies of this nature, (7) of this nature but in subsequent research the sample size should be larger.

The intention is to conduct an extended study using the results from this preliminary study. The follow-up study will have a much larger sample size and include more puzzles. A consideration for the puzzles is to vary the orientation, length and position of the target word as well as including distractor words in some of the presented puzzles. Distractors should only influence the search if the word can be recognized, hence having very little impact on the search in L3.

Additionally, since spacing could play a vital role in the word recognition process, the spacing between rows and columns can be varied in order to determine whether previous findings on reading carry over to a word puzzle search.

This study presented participants with a simple word search puzzle containing either a single long or short target word in one of three languages. Overall, the language and word length had very little effect on gaze behavior. Fixations were found to be shorter than typical reading, this could be as a result of the nature of the task or the increased spacing between words.

It appears that the search strategy is the factor most influenced by the puzzle. The search strategy employed is a personal choice of the participant, with many preferring a random search. There is however evidence to suggest that the search pattern changes as participants are tasked to find a word in a language they are less fluent in (or not at all).

In conclusion, the study found gaze patterns were not influenced by language or length of word but that in all instances, participants employ a search strategy based on the word to be found and the language presented.

### Ethics and Conflict of Interest

The author(s) declare(s) that the contents of the article are in agreement with the ethics described in http://biblio.unibe.ch/portale/elibrary/BOP/jemr/ethics.html and that there is no conflict of interest regarding the publication of this paper
